# Microbial Diversity of Pinnacle and Conical Microbial Mats in the Perennially Ice-Covered Lake Untersee, East Antarctica

**DOI:** 10.3389/fmicb.2020.607251

**Published:** 2020-12-10

**Authors:** Carla Greco, Dale T. Andersen, Ian Hawes, Alexander M. C. Bowles, Marian L. Yallop, Gary Barker, Anne D. Jungblut

**Affiliations:** ^1^Department of Life Sciences, Natural History Museum, London, United Kingdom; ^2^School of Biological Sciences, University of Bristol, Bristol, United Kingdom; ^3^Carl Sagan Center, SETI Institute, Mountain View, CA, United States; ^4^Coastal Marine Field Station, University of Waikato, Tauranga, New Zealand; ^5^School of Life Sciences, University of Essex, Colchester, United Kingdom

**Keywords:** 16S rRNA gene, 18S rRNA gene, microbial mat, pinnacles, cones, stromatolite, lake, Antarctica

## Abstract

Antarctic perennially ice-covered lakes provide a stable low-disturbance environment where complex microbially mediated structures can grow. Lake Untersee, an ultra-oligotrophic lake in East Antarctica, has the lake floor covered in benthic microbial mat communities, where laminated organo-sedimentary structures form with three distinct, sympatric morphologies: small, elongated cuspate pinnacles, large complex cones and flat mats. We examined the diversity of prokaryotes and eukaryotes in pinnacles, cones and flat microbial mats using high-throughput sequencing of 16S and 18S rRNA genes and assessed how microbial composition may underpin the formation of these distinct macroscopic mat morphologies under the same environmental conditions. Our analysis identified distinct clustering of microbial communities according to mat morphology. The prokaryotic communities were dominated by Cyanobacteria, Proteobacteria, Verrucomicrobia, Planctomycetes, and Actinobacteria. While filamentous *Tychonema* cyanobacteria were common in all mat types, *Leptolyngbya* showed an increased relative abundance in the pinnacle structures only. Our study provides the first report of the eukaryotic community structure of Lake Untersee benthic mats, which was dominated by Ciliophora, Chlorophyta, Fungi, Cercozoa, and Discicristata. The eukaryote richness was lower than for prokaryote assemblages and no distinct clustering was observed between mat morphologies. These findings suggest that cyanobacterial assemblages and potentially other bacteria and eukaryotes may influence structure morphogenesis, allowing distinct structures to form across a small spatial scale.

## Introduction

Microbial mat communities dominated by cyanobacteria are important ecosystem components across a diverse range of marine and freshwater environments and form the oldest identifiable fossil assemblages from the Earth’s early biosphere (Allwood et al., 2006). Modern environments with low disturbance and an absence of metazoan grazers, for example hot springs (Ward et al., 1998; Pepe-Ranney et al., 2012) and hypersaline environments (Burns et al., 2004; Dupraz and Visscher, 2005; Goh et al., 2009), are therefore of particular interest as these conditions allow microbial mats to produce complex organo-sedimentary structures. These morphologies range from small scale ridges and peaks to large-scale 3D structures with complex topographies, and may provide insights into how ancient stromatolites were formed (Walter et al., 1976; Hawes et al., 2019).

In Antarctic perennially ice-covered lakes, multi-layered, three-dimensional structures form that are often laminated through annual growth (Hawes et al., 2001) and trapping and binding of sediment (Sutherland and Hawes, 2009). Well-studied examples come from the McMurdo Dry Valleys lake systems, an array of lakes with unique physical and chemical properties where thick microbial mats cover the floors of the lakes within the photic zone. These structures encompass a diverse range of morphologies from flat prostrate and pinnacle mats in Lake Hoare (Sutherland and Hawes, 2009; Hawes et al., 2016), branched stromatolites in Lake Joyce (Mackey et al., 2015) and cuspate pinnacles in Lake Vanda (Sumner et al., 2016), and their microbial assemblages have been studied with both microscopy and molecular methods. Mat topography is often linked to environmental conditions driving biological processes such as metabolism, growth and biomass accumulation. Zonation of mat morphologies have been reported along environmental gradients in lake environments with heterogeneous profiles, underpinned by changes in cyanobacterial and bacterial composition driven by local habitat conditions such as irradiance, oxygen and sulfide concentrations (Jungblut et al., 2016), sedimentation rates or carbonate precipitation (Mackey et al., 2018). Our current knowledge of the mechanisms that initiate and maintain the growth of microbially mediated structures is currently incomplete due to the complexity of processes and microbial interactions occurring within these ecosystems. Models for the initiation of pinnacle structure morphogenesis include the initiation of growth from random irregularities in prostrate flat mats followed by biomass accrual over time and the colonization by a more diverse community of cyanobacteria (Sumner et al., 2016), and the growth of cyanobacteria around trapped photosynthetic oxygen bubbles within mats and subsequent lithification (Bosak et al., 2009, 2010).

Andersen et al. (2011) reported novel cyanobacteria-based microbial mats in Lake Untersee, East Antarctica. Benthic mats dominate the environment in Lake Untersee to a depth of at least 130 m, and these mats form two distinct macroscopic structures in addition to flat mats: cuspate pinnacles and decimetre-scaled, laminated conical structures. Whilst pinnacle structures are common throughout Antarctic ecosystems (Sumner et al., 2016), the large complex cones in Lake Untersee are the first report of such structures in a modern environment. These two morphologically different structures occur adjacent to each other within the same environmental conditions, rather than along an environmental gradient as in other Antarctic lakes (Jungblut et al., 2016). This suggests that distinct morphologies can occur within homogeneous environmental conditions meaning that the processes underlying structure development are more complex than predictable responses to geochemical variables and may involve species-specific microbial behaviors.

Stromatolites are defined as internally laminated sedimentary structures produced by sediment trapping, binding or precipitation as a result of growth and metabolic activity of micro-organisms, in particular Cyanobacteria (Grey and Awramik, 2020). The microbial structures in Lake Untersee are examples of modern unlithified stromatolites and have a similar morphology to Archean and Proterozoic conical stromatolites, in particular the Early Archean large conical stromatolites of the Strelley Pool Formation in Western Australia (Allwood et al., 2007). Modern, actively growing stromatolites have been shown to be useful analogs for fossil stromatolites, and can provide a basis to understand the conditions which on Early Earth enabled the formation of similar structures of potential biological origin (Jones et al., 2002; Allwood et al., 2006; Mackey et al., 2015; Grey and Awramik, 2020). Additionally, studying the community structure of modern stromatolites in Antarctic lakes would improve our understanding of the interactions between microbial communities and the environment of Early Earth ecosystems (Mackey et al., 2015). Few studies have described the cyanobacterial and bacterial communities of Lake Untersee through microscopy and next-generation sequencing (Andersen et al., 2011; Koo et al., 2017; Weisleitner et al., 2019) with no characterisation of the richness and composition of 18S rRNA gene eukaryotic assemblages to date. A 16S rRNA gene and metagenomic sequencing analysis of a single conical structure identified distinct communities in surface, middle and lower laminae, with a transition to more heterotrophic taxa occurring with depth within the mat (Koo et al., 2017). Although differences in cyanobacterial communities between cones and pinnacles have been reported through microscopy observations of cyanobacteria, no study has compared the community composition of the different morphological structures using molecular methods. To address these knowledge gaps, the present study describes the community composition of prokaryotes and eukaryotes from microbial mats from Lake Untersee through 16S and 18S rRNA high-throughput sequencing. We compared the microbial communities of flat, pinnacle and conical mat morphologies and evaluated the association of microbial community assemblage and macroscopic microbial mat structure in the upper laminations associated with photosynthetic carbon as documented in Andersen et al. (2011).

## Materials and Methods

### Study Site

**FIGURE 1:**
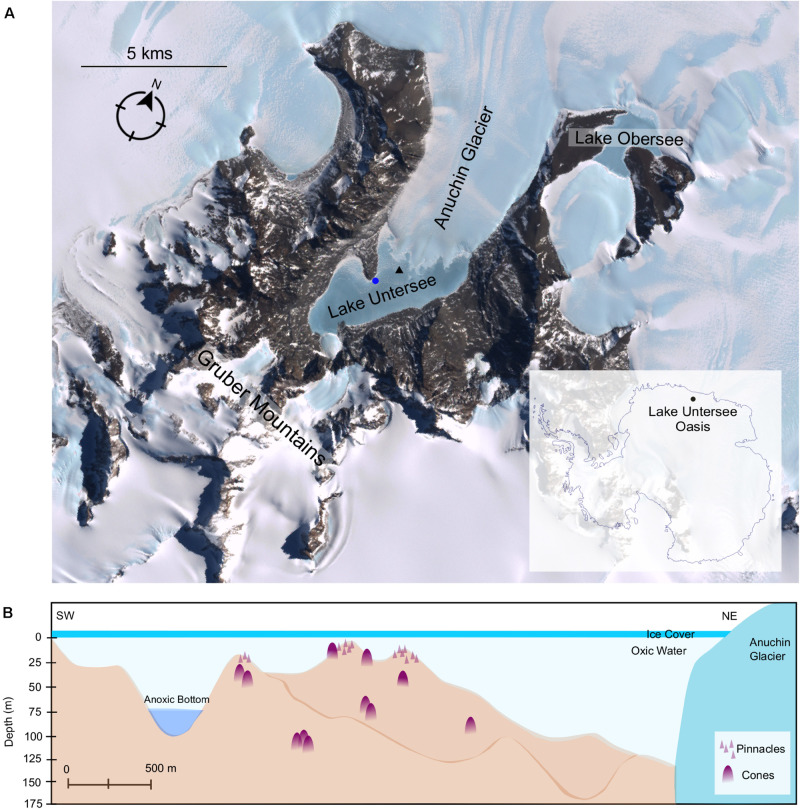
**(A)** Satellite imagery of Lake Untersee Oases. Image capture by Landsat 7 satellite (download via LIMA database from United States Geographical Survey). Benthic sampling and water profiling sites are denoted by a blue circle and black triangle, respectively. **(B)** Schematic illustration of Lake Untersee based on Wand et al. (2006) and (Faucher et al., 2019). Representative symbols for pinnacles and cones as shown in Figure 2, which are not to scale.

Lake Untersee (Figure 1A) is the largest surface lake in Queen Maud Land (area of 8.73 km^2^). It is located at 71° 20.736′S, 13° 27.842′E in the Otto-von-Gruber-Gebirge (Gruber Mountains) and lies adjacent to the Anuchin Glacier (Faucher et al., 2020). The lake is ultra-oligotrophic, 169 m at maximum depth and has likely retained its ice cover during the last 300–500 years without receiving additional input from surface streams (Wand and Perlt, 1999; Faucher et al., 2020). The ice cover thickness ranges between 3.8 and 2.2 m (average 3.0 m) (Wand et al., 1997). The lake is primarily fed by subaqueous terminus melting of the Anuchin Glacier and subglacial meltwater and has no outlet. The lake is composed of two sub-basins separated by a sill at approximately 50 m depth (Figure 1B). The smaller basin to the south is 100 m deep and stratified with an anoxic base. The larger, northern basin is 169 m deep and is well-mixed, close to isothermal and oxygenated to the bottom (Wand et al., 1997). The larger basin is characterized by high pH values (9.8–10.6), supersaturation with dissolved oxygen (up to 150%) and low primary production of phytoplankton. The high pH has been attributed to weathering of the predominant anorthosite rock in the lake’s catchment (Kaup et al., 1988; Andersen et al., 2011). The presence of a perennial ice-cover has a profound effect on the physical and biological properties of lakes (McKay et al., 1994; Hawes et al., 2019). Approximately 5% of surface irradiance is transmitted by the lake ice, and the vertical extinction coefficient for scalar photosynthetically active radiation (PAR) was determined as 0.033 m^–1^ with an estimation of 3, 1, and 0.1% of surface irradiance reaching the depths of 20, 42, and 145 m depth, respectively (Andersen et al., 2011). It has been shown that for ice-covered lakes in other parts of Antarctica the ice cover selectively transmits blue-green wavelengths with almost no transmission of light above a wavelength of 650 nm, with the blue-green bias, and absence of red and far-red light, reinforced by the absorption characteristics of the water column (Hawes and Schwartz, 2001). The perennial ice-cover at Lake Untersee is essentially opaque at wavelengths 680 nm and beyond (*T* < 0.01%) with a steep drop-off at a depth of 20 m for wavelengths beyond 580 nm (Andersen, unpublished data).

**FIGURE 2:**
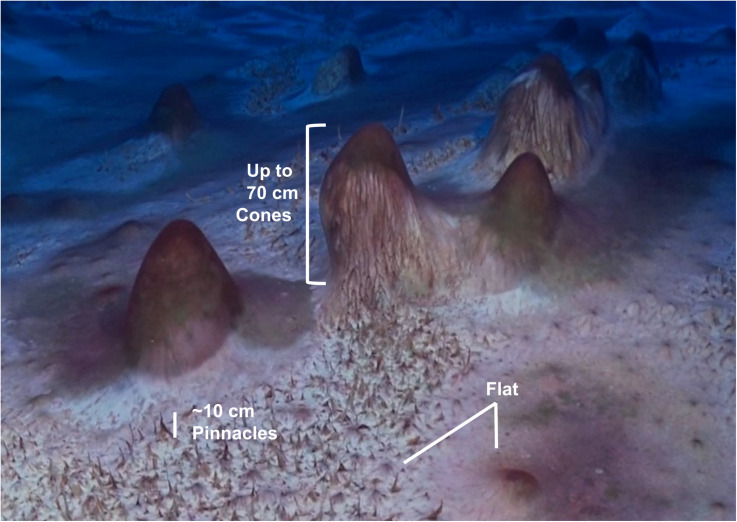
Cones, pinnacles and flat cyanobacteria-based microbial mats with purple and green pigmentation due to phycoerythrin and chlorophyll, respectively, grow in the perennially ice-covered Lake Untersee, East Antarctica.

The lake floor is covered with photosynthetic benthic microbial mats dominated by filamentous cyanobacteria morphotypes. In addition to flat mats, the benthic mats have two categories of macroscopic structures: pinnacle and conical structures (Figure 2). The pinnacles have a narrow, elongated structure and are 1–1.5 cm in diameter and up to 15 cm tall. The cones are large structures with rounded tops, heights ranging up to 70 cm and a diameter range from 10 to 60 cm. They are composed of a thin microbial mat covering a soft, laminated structure of fine clay sediment, with purple pigmentation (caused by the cyanobacterial light-harvesting pigment phycoerythrin) concentrated at the apex of the cones (Andersen et al., 2011). The sediments are composed of fine clay minerals (1 μm in size) with occasional larger feldspar crystals (up to 50 μm). The feldspar was shown to be Ca plagioclase, and the clays minerals typically had a Al:Si:O mole ratio of 1:2:5–6 (Andersen et al., 2011). The organic C abundances of the microbial mats is 10.14% in the upper pigmented sections (0 – 0.5 cm) (Andersen, unpublished data) and range from 1.0 to 5.8 % in the lower laminations, decreasing with depth within the mats (Marsh et al., 2020). Microbial mats have been observed to extend from 8 to 130 m deep in the lake; pinnacles were observed at depths up to around 20 m whilst cones were observed regularly throughout the lake to depths of 130 m (Figure 1B). Previous studies have confirmed the photosynthetic competence of the microbial mats through *in situ* Pulse Amplitude Modulated (PAM) Fluorometry (Andersen et al., 2011) and net oxygen production during summer through microelectrode profiling (Hawes et al., 2019).

### Water Column Profile

Water profiling was undertaken from holes drilled through the ice cover in the northern basin of the lake in November 2011 (austral summer). Temperature, pH, chlorophyll-*a* and dissolved oxygen were profiled using a freshly calibrated Hydrolab DS5 multi-parameter water quality sonde (OTT Hydromet, 5600 Lindbergh Drive, Loveland, CO 80539, United States) connected to a field-portable computer.

### Microbial Mat Sample Collection

Benthic microbial mats were collected by scientific divers utilizing SCUBA from a location in the southern basin of Lake Untersee in November 2011 (austral summer) using a sterile blade and collected into sterile 50 ml conical tubes from a depth of 20 m. Three distinct macroscopic mat morphologies were sampled: flat, cone and pinnacle shaped microbial mats (Figure 2). Additionally, a loose mesh of darkly pigmented cyanobacteria filaments from the top of the microbial mats were collected. The flat mat samples were collected between cones and between pinnacles. Following transfer of the mat samples to the field laboratory the pigmented upper sections of the microbial mats (laminae less than 1 mm in thickness) were separated from lower non-pigmented sediment rich portion of the microbial structures using a sterile blade and rinsed in sterile deionized water for DNA extraction. The samples were stored at −20°C after collection and permanently stored at −80°C at the Natural History Museum until further processing.

### DNA Extraction, Polymerase Chain Reaction (PCR) and Next-Generation Sequencing (NGS)

Between 0.05 and 0.30 g of microbial mat material from the pigmented upper sections was used for DNA extraction from each sample using the PowerBiofilm DNA Isolation Kit (MO BIO Laboratories, Carlsbad, CA, United States) following the manufacturer’s protocol. Duplicate DNA extractions were performed for each sample and pooled to reduce sampling bias. The extracted genomic DNA was PCR-amplified in triplicate using 515f-806r primers (Caporaso et al., 2012) and 1391f-EukBr primers (Amaral-Zettler et al., 2009; Caporaso et al., 2012). The 16S rRNA and 18S rRNA gene PCR products were cleaned using AxyPrep Mag PCR clean-up (Axygen, New York, NY, United States), quantified using a Qubit fluorometer (Introgen, United Kingdom) and pooled prior to sequencing. Sequencing was performed on the Illumina MiSeq platform by the Natural History Museum sequencing facility. The high-throughput sequencing data and associated metadata from this study were submitted to the Sequence Read Archive (SRA) in GenBank under BioProject PRJNA638357.

### Sequence Analysis

Raw sequences were processed in QIIME2 (version 2018.8) (Bolyen et al., 2019) using the dada2 package (Callahan et al., 2016, 2019). Sequences were quality filtered, merged and the dada2 algorithm was applied to denoise sequences and generate an Amplicon Sequence Variant (ASV) table. Low abundance and low-quality sequences were filtered. Taxonomy was assigned using the q2-feature-classifier (Bokulich et al., 2018) classify-sklearn naïve Bayes taxonomy classifier against the SILVA 132 database (Quast et al., 2013; Yilmaz et al., 2014). All mitochondrial and chloroplast plastid 16S rRNA gene sequences based on this classification were removed from the 16S rRNA gene data set, and any bacterial sequences amplified due to unspecific binding of the primers were removed from the 18S rRNA data set. Statistical analysis was conducted in R using Vegan (version 2.5-6) (Oksanen et al., 2019) and Phyloseq packages (version 1.26.1) (McMurdie and Holmes, 2013). Rarefaction was not performed to avoid an inflated rate of false positives (McMurdie and Holmes, 2014). Metazoa were removed from diversity and community structure analyses. Alpha diversity was tested using Observed, Shannon’s and InvSimpson, and differences between diversity indices were tested with an ANOVA and Tukey’s HSD post-hoc test. Non-metric multidimensional scaling (nMDS) was performed using Bray-Curtis distance on the (relative abundance transformed) ASV table, and an analysis of similarities (ANOSIM) was carried out with 999 permutations.

### Phylogenetic Mapping of Cyanobacterial ASVs Using Evolutionary Placement Algorithm (EPA)

A reference cyanobacterial tree was constructed using 42 sequences including the closest matches to the SILVA database for each cyanobacterial ASV. Additionally, cyanobacterial 16S rRNA gene sequences from published Arctic and Antarctic studies and references strains of the identified morphotypes were also included in the phylogenetic tree for comparison. The 16S rRNA gene sequence of *Gloeobacter violaceus* PCC 7421 (NR074282) was used as the outgroup for phylogenetic analysis. The reference sequences were aligned using ClustalX (version 2.1) to produce a 640 bp multiple sequence alignment, and a reference Maximum-likelihood (ML) phylogenetic tree was constructed in RaxML (version 8.2.12) (Stamatakis et al., 2008) using the GTRCAT substitution model for the bootstrapping phase and the GTRGAMMA for the final tree inference. A best-scoring ML tree was obtained based on 1000 bootstraps. The cyanobacterial ASVs obtained in this study were aligned using PaPaRa 2.5 (Berger and Stamatakis, 2012) and mapped onto the reference tree using the Evolutionary Placement Algorithm (EPA-NG) (Barbera et al., 2019) and only read placements with likelihood weight of >0.5 were retained.

## Results

### Water Column Profiles

**FIGURE 3:**
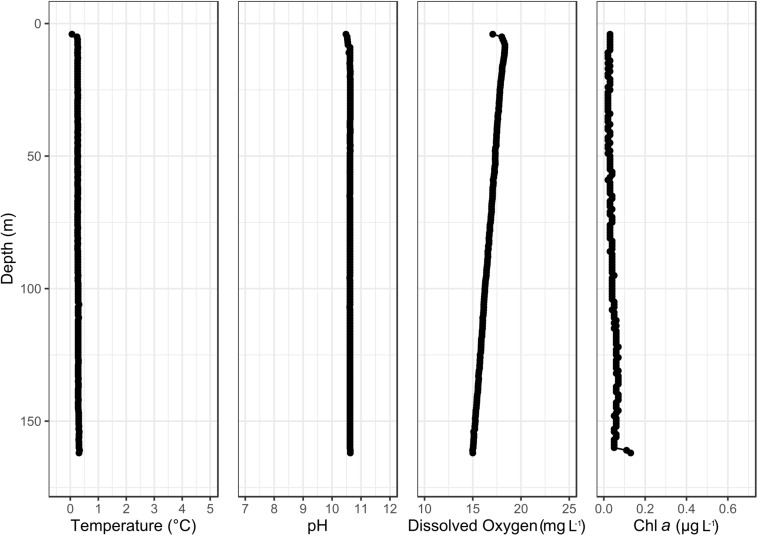
Temperature, pH, dissolved oxygen and chlorophyll-*a* water column profile of the northern basin of Lake Untersee.

The northern basin of Lake Untersee had a mixed water column with a pH 10.5 and temperature of 0.3°C. The dissolved oxygen ranged from 18 mg/l under the ice to 15 mg L^–1^ at 150 m, and planktonic chlorophyll-*a* fluorescence confirmed a low concentration below 0.2 μg L^–1^ across all depths (Figure 3).

### 16S and 18S rRNA Gene Composition of Microbial Mats in Lake Untersee

**FIGURE 4:**
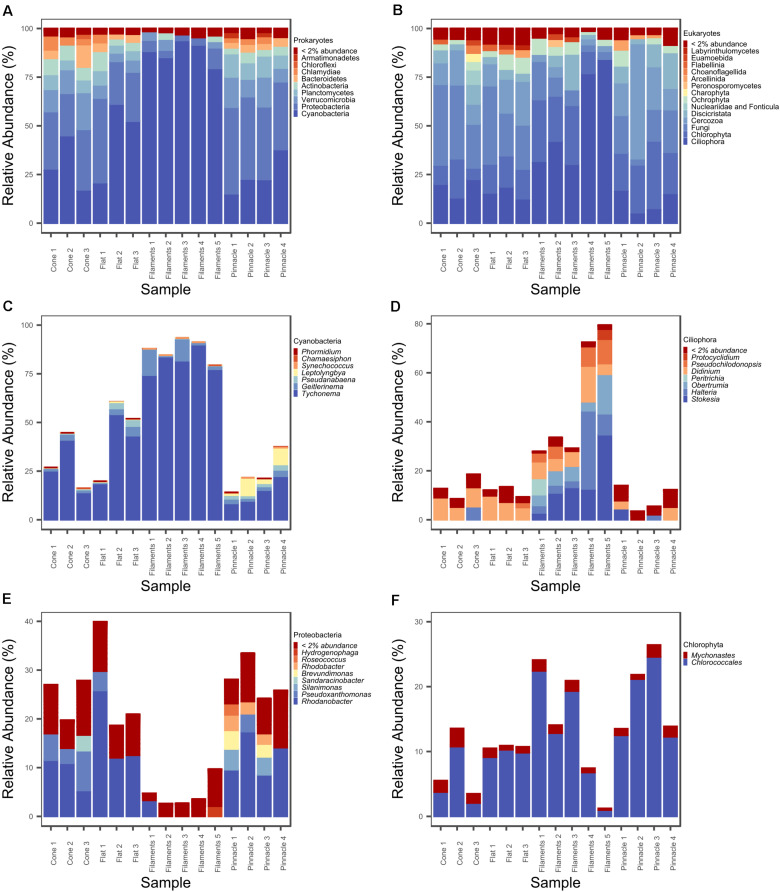
Relative abundance (%) plot of **(A)** prokaryotic phyla, **(B)** eukaryotic major groupings, **(C)** cyanobacterial genera, **(D)** ciliophoran genera, **(E)** proteobacterial genera, and **(F)** chlorophyte genera.

**FIGURE 5:**
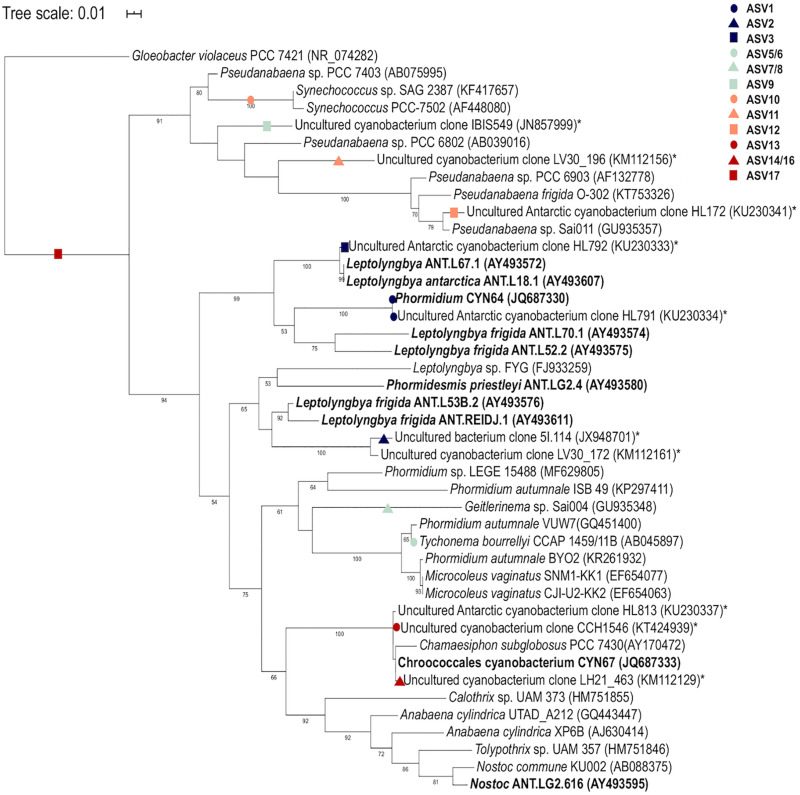
Phylogenetic mapping analysis of Lake Untersee cyanobacterial ASVs. Mapping of sequences from this study are denoted by colored shapes. Environmental sequences from Antarctic environments are denoted by asterisk (*) and Antarctic strains are denoted in bold. Bootstrap values >50% are labeled.

A total of 360 ASVs were identified in the 16S rRNA gene data set and 150 were identified in the 18S rRNA dataset from 1,869,632 to 654,807 sequences from the 15 microbial mat samples, respectively. The five most prevalent bacterial phyla in the Lake Untersee samples (accounting for 91.71% of the total assigned ASVs across samples) were Cyanobacteria (49.7%), Proteobacteria (23.6%), Verrucomicrobia (8.8%), Planctomycetes (4.9%) and Actinobacteria (4.2%) across filament mesh, flat, cone and pinnacle mat samples (Figure 4A). No archaeal ASVs were identified across all samples in this study. 120 Proteobacteria ASVs were identified and the most abundant genus represented was *Rhodanobacter* (Figure 4E). 17 cyanobacterial ASVs were present in the Lake Untersee microbial mats. The dominant cyanobacterial genus, *Tychonema*, represented 87.5% of the relative abundance of all cyanobacterial ASVs (Figure 4C). *Tychonema* as well a *Pseudanabaena* (2.3%) and *Leptolyngbya* (1.9%) were recognized in all fifteen communities. *Leptolyngbya* was identified in the pinnacle samples at a higher proportion and contributed 20.1% of cyanobacterial relative abundance in pinnacle samples. ASVs assigned to *Leptolyngbya* were further identified as *Leptolyngbya antarctica, Leptolyngbya* sp. ULC077 and *Leptolyngbya* sp. LCR-CYANT35 (Supplementary Table 3). Phylogenetic mapping of the 17 cyanobacterial ASVs confirmed the taxonomic assignment by SILVA to cyanobacterial genera (Figure 5). The evolutionary mapping analysis showed that several ASVs were related to environmental sequences and cyanobacteria strains previously identified from microbial mats in Antarctica including Pyramid Trough and the McMurdo Dry Valley Lake Hoare and Lake Vanda (Southern Victorialand). In particular, *Chamaesiphon* groups to Lake Hoare, *Pseudanabaena* to Pyramid Trough and *Leptolyngbya* to Lake Hoare and Lake Vanda.

Our 18S rRNA gene analyses identified ASVs from several eukaryotic supergroups in all 15 samples from filament mesh, flat, pinnacle, and cone shaped macroscopic mat morphologies. The eukaryote assemblages were dominated by Ciliophora (supergroup Alveolata, 31.7%), Chlorophyta (supergroup Archaeplastida, 19.7%), Fungi (supergroup Opisthokonta, 19.6%), Cercozoa (supergroup SAR, 15.2%) and Discicristata (Excavata, 7.5%) across filament mesh, flat, cone and pinnacle mat samples (Figure 4B). 32 ciliate ASVs were identified, largely comprised of *Stokesia, Halteria, Didinium*, and *Obertrumia* (Figure 4D). There were also four ASVs identified as Chlorophyta with *Mychonastes* and *Chlorococcales* (further identified as *Chlorococcales* sp. VII3) being most abundant (Figure 4F). A range of fungi were detected grouping within the phyla Ascomycota, Chytridiomycota and Cryptomycota. In addition, other protists were detected grouping with Arcellinid testate amoebae, Choanoflagellates, Ochrophyta, and Labyrinthulomycetes. The 18S rRNA gene data also showed that the microbial mats contain metazoa in Lake Untersee, and ASVs were identified as rotifers (bdelloidea, class Adinetida) and Monogononta (Flosculariacea and Ploimida), and nematodes Dorylaimida and Chromadorea (Araeolaimida and Monhysterida). A complete list of genus level taxonomic assignments for 16S and 18S rRNA gene ASV are provided in Supplementary Tables 1, 2.

### Comparison of 16S and 18S rRNA Gene Communities in Pinnacle, Cone and Flat Microbial Mats in Lake Untersee

**FIGURE 6:**
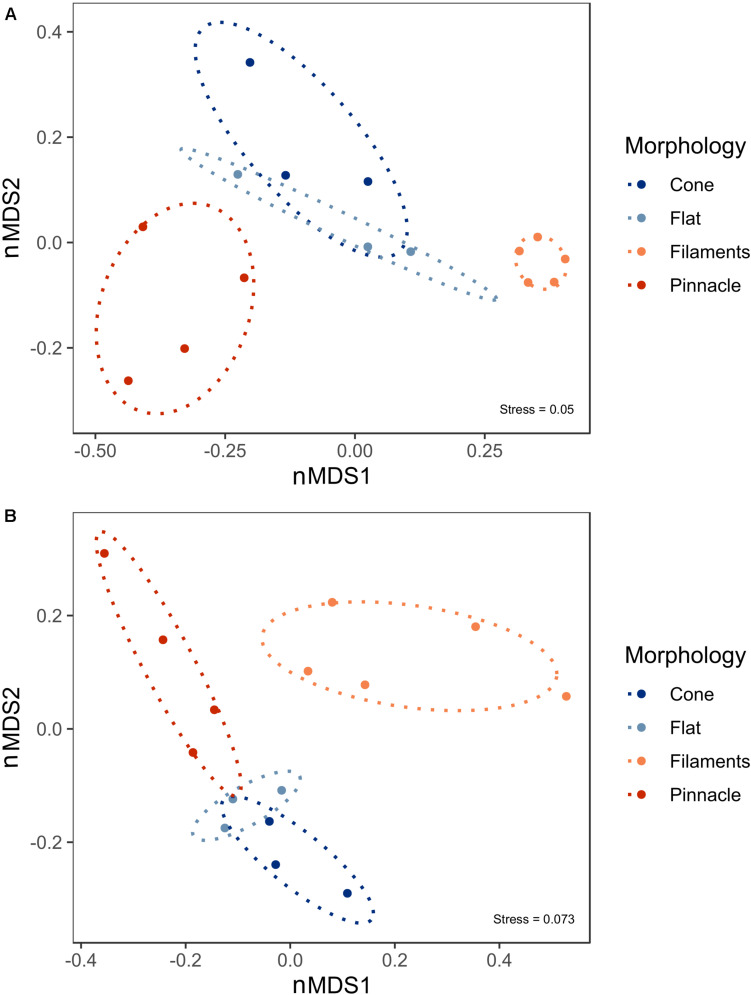
Non-metric Multidimensional scaling (nMDS) plot derived from standardized ASV abundance for **(A)** prokaryote and **(B)** eukaryote communities in 15 benthic microbial mat samples from Lake Untersee. Ellipses represent 95% confidence intervals.

Microbial mats cover the photic zone of the bottom of Lake Untersee, and pinnacle and conical shaped 3-dimensional macroscopic structures develop from the flat microbial mats. nMDS of the 16S rRNA gene prokaryotic communities from the pigmented laminae of the different microbial mat morphologies showed a distinct clustering of samples by microbial mat type. There was a significant difference between the prokaryotic communities forming the loose filament mesh and pinnacle versus the cone and flat mat morphologies (Figure 6A, ANOSIM: 999 permutations, *p* = 0.001). However, the flat and cone communities were not significantly different. A significant difference was observed in the 18S rRNA gene eukaryotic community structure and distinct clustering of the loose filament mesh was identified (Figure 6B, ANOSIM:999, *p* = 0.001). However, the eukaryote communities in the flat, pinnacle and cone eukaryotic communities did not demonstrate distinct clustering and showed high variability between individual samples.

**FIGURE 7:**
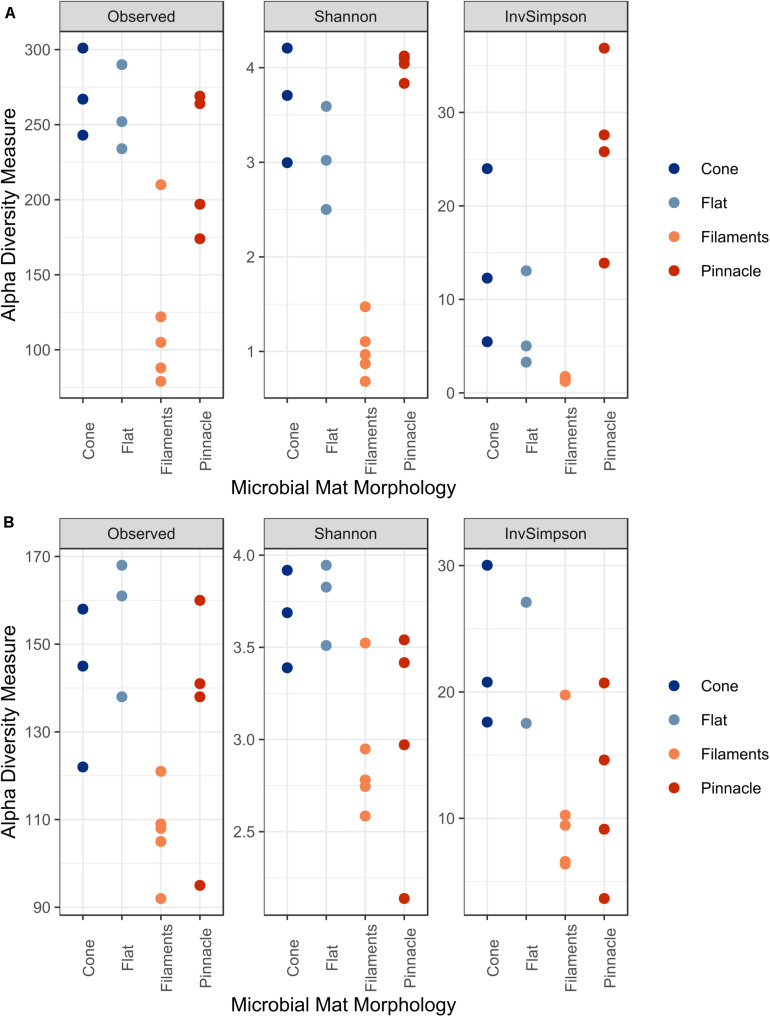
The alpha diversity indices Observed, Shannon and InvSimpson for **(A)** 16S and **(B)** 18S rRNA datasets.

There was a significant difference in alpha diversity for Observed (*F* = 10.14, *p* = 0.0017), Shannon (*F* = 50.81, *p* < 0.001) and InvSimpson (*F* = 9.79, *p* = 0.0019) for the 16S rRNA communities across the four different microbial mat types (Figure 7A). The three diversity indices were significantly different in pairwise comparison between filament mesh versus cone (Observed *p* = 0.0032, Shannon *p* < 0.001), flat (Observed *p* = 0.0059, Shannon *p* < 0.001) and pinnacle mats (Observed *p* = 0.019, Shannon *p* < 0.001, InvSimpson *p* < 0.001). However, the diversity indices varied between samples for mat types and, therefore, no significant differences were identified between cone, pinnacle and flat mat communities. Alpha diversity varied between replicate samples also for 18S rRNA gene communities for mat types and no significant differences were identified between filament mesh, pinnacle, cone and flat mat morphologies (Figure 7B).

## Discussion

### Diverse Prokaryotic and Eukaryotic Communities in Pinnacle, Conical, and Flat Shaped Microbial Mats in Lake Untersee

The benthic microbial mats of the southern basin of Lake Untersee were dominated by Cyanobacteria, but also harbored a diverse community including Proteobacteria, Verrucomicrobia, Planctomycetes, and Actinobacteria. The findings of this study correlate with previous reports on the prokaryotic community in benthic microbial mat structures from Lake Untersee (Andersen et al., 2011; Koo et al., 2017; Weisleitner et al., 2019). Although this lake does not form a meltwater moat during the summer, it has been shown that microbial diversity in cryoconite holes on the Anuchin glacier may play a role as a source for inocula of prokaryotic biota into Lake Untersee through glacial meltwater (Weisleitner et al., 2019).

Cyanobacteria, shown to be a dominant part of the community of Lake Untersee, are important phototrophs in polar freshwater and terrestrial ecosystems, particularly habitats in Antarctica (Vincent et al., 2000; Chrismas et al., 2015). *Tychonema*, *Leptolyngbya*, and *Pseudanabaena* were identified in all benthic mat structures. Morphotypes identified by microscopy as *Leptolyngbya* and purple pigmented *Phormidium* were described in pinnacles and cones for Lake Untersee (Andersen et al., 2011). The most abundant cyanobacterial ASV was classified as *Tychonema* sp. based on the SILVA reference database, and was closely related to *Phormidium autumnale* within a poorly resolved *Phormidium* clade based evolutionary phylogenetic mapping analyses (Strunecký et al., 2013). *P. autumnale* and *Tychonema* sp. have a similar morphology based on light microscopy, and it is therefore likely that the *Tychonema* identified in this study based on short 16S rRNA gene sequences and the SILVA reference database is the *P. autumnale* morphotype identified to be very abundant in Andersen et al. (2011). *P. autumnale* (recently revised as *Microcoleus*) is a motile species which moves using a gliding motion (Hoiczyk and Baumeister, 1995; Strunecký et al., 2013). Three ASVs were assigned to *Leptolynbya* sp. which were further identified as *L. antarctica*, *Leptolyngbya* sp. ULC077 and *Leptolyngbya* sp. LCR-CYANT35 (Supplementary Information). These strains are within two different clades of *Leptolyngbya* (Chrismas et al., 2015). A high number of ASVs found in this study mapped to sequences from Antarctic lake environments, including the McMurdo Dry Valley Lakes, despite a wide geographic separation. These similarities suggest a potential wide dispersal of broadly tolerant cyanobacteria within Antarctic freshwater environments that are particularly successful in under-ice environments (Zhang et al., 2015) which may occur via mechanisms of long-range transport (Hughes et al., 2004; Muñoz et al., 2004).

Proteobacteria comprised the second most abundant phylum in all samples which is a common feature of Antarctic microbial mat environments. *Rhodanobacter*, a gram-negative rod-shaped bacterium, was identified as the most abundant genus of Proteobacteria, and has been identified previously in many Antarctic environments (Li et al., 2019). No ASVs assigned to archaea are reported in this study. It is possible that archaea present in the samples were undetected, as the 515f-806r primers used in this study have been shown to underrepresent certain archaeal taxa (Parada et al., 2016). Archaea identified as Methanomicrobia and Thermoplasmata have previously been reported in benthic samples from Lake Untersee using specific archaea 16S rRNA gene primers (Weisleitner et al., 2019). Additionally, a methanogenic archaea in the family *Methanoregulaceae* has been isolated from the anoxic sediments of the smaller, southern basin (Wagner et al., 2019). However, archaea have previously been reported in low abundances in certain Arctic and Antarctic microbial mats using 16S rRNA gene sequencing and metagenomic methodologies (Brambilla et al., 2001; Varin et al., 2010, 2011; Jungblut et al., 2016).

Another limitation of the study is the use of DNA which may amplify relic DNA in the biofilms and planktonic organisms from the surrounding water column. Additionally, the cold, alkaline conditions may preserve DNA fragments over long time scales. However, the here identified cyanobacterial communities using 16S rRNA gene sequencing have good overlap with the previously identified morphotypes (Andersen et al., 2011), which suggest a satisfactory coverage of the cyanobacterial-mat communities. Furthermore, this contamination may result in a biased representation of the community structure and may impact the detection of rare ASVs, other studies have demonstrated that, despite its abundance, relic DNA has a minimal effect on estimates of taxonomic and phylogenetic diversity (Lennon et al., 2018).

This study is the first high-throughput sequencing report on eukaryote diversity and community assembly of microbial mats in Lake Untersee. The microbial eukaryotic community structure was heterogenous in diversity between mat structures and had a lower richness than the prokaryotic communities. The most abundant ASV within Ciliophora was assigned as *Stokesia* sp. This genus has been reported in other Antarctic environments such as cryoconites (Sommers et al., 2018, 2019). *Stokesia* sp. are reported mixotrophs (Amblard et al., 1993), combining phototrophy and phagotrophy, a tactic which may present an advantage when resources are scarce (Marshall and Laybourn-Parry, 2002). Mixotrophic ciliates have been reported in a number of Antarctic lakes (Gerea et al., 2013) and several studies support the idea that mixotrophs are as important bacterivorous grazers as heterotrophs in oligotrophic systems (Unrein et al., 2007; Gerea et al., 2013). Additionally, *Didinium, Peritrichia*, and *Obertrumia* have been reported in McMurdo Dry Valley lakes (Roberts et al., 2000; Xu et al., 2014).

The eukaryotic community structure was mainly heterotrophic, and only 20% of the relative abundance was Chlorophyta with only four ASVs representing photosynthetic taxa. Contrastingly, Cyanobacteria composed nearly 50% of prokaryotic community across the samples, consolidating their importance as the principal photosynthesising group in this environment. Dominance of benthic phototrophic communities by filamentous cyanobacteria in Antarctic perennially ice-covered lakes is well documented (Wharton et al., 1983). The benthic phototrophs are adapted to optimize the use of the available low, blue-green irradiance (Hawes and Schwartz, 2001; Hawes et al., 2014). The seasonal variability of PAR means that growth cessation and potentially the formation of resting stages occurs during the prolonged periods of darkness during winter, and this cyclic growth is recognizable as annual laminations in the mat structure (Hawes et al., 2001). The most abundant ASV within Chlorophyta was assigned within the clade Chlorococcales, and linked to an isolate from Lake Fryxell *Chlorococcales* sp. *VII3* (De Wever et al., 2009). A key aspect of the eukaryotic community structure is the confirmation of an absence, or very low abundance, of diatoms, as reported through microscopy by Andersen et al. (2011). Diatoms are often found in other Antarctic lakes and are particularly prominent in Dry Valley lake microbial mat communities. For example, a diverse community of diatoms including, *Navicula*, *Nitszchia*, and *Diadesmis* have been reported in lakes Hoare (Spaulding et al., 1997), Fryxell (Jungblut et al., 2016) and Vanda (Sumner et al., 2016). In Lake Untersee, the paucity of diatoms has been linked to the extreme high pH (Andersen et al., 2011), which has been linked to weathering of clay minerals in an ice-sealed water body (Wand et al., 1997; Andersen et al., 2011; Marsh et al., 2020). High pH extends many millimeters into the mat surface in Lake Untersee (Hawes et al., 2019) and this has been shown to constrain growth in diatoms (Chen and Durbin, 1994), although there are examples of diatoms tolerant of high pH (Sutherland, 2009).

The findings highlight that cyanobacterial mats are important microhabitats for eukaryotic organisms, especially for heterotrophic protists and fungi, as they are environments rich in inorganic nutrients (Bonilla et al., 2005), recycled organic matter and bacteria (Varin et al., 2010). Cyanobacteria and bacteria may provide a food source for grazing bacterivorous heterotrophs such as ciliates and the metazoan meiofauna resulting in an increased trophic complexity within the mats (Duffy and Stachowicz, 2006) where chemical signaling and species interactions are likely important (Pernthaler, 2005; Martinez-garcia et al., 2012; Velázquez et al., 2017). Future studies focusing on the functional genes underlying the metabolic processes within the microbial mats and genome sequencing of key community members may help elucidate the complex processes and organism interactions that shape the community and contribute to structural morphogenesis.

### Comparison of Microbial Communities in Pinnacle and Conical Mats and Implications for Structure Formation

Flat, pinnacle and conical mat structures were shown to have similar richness, apart from the loose mesh of filaments above the mat which had a significantly lower number of prokaryotic ASVs. This reduced richness can be attributed to the increased relative abundance of Cyanobacteria and lower relative abundance of Proteobacteria, Actinobacteria, and Verrucomicrobia. Changes in community composition across stratified laminae were previously reported in a cone from Lake Untersee which was dissected into three distinct layers (Koo et al., 2017). A decrease in relative abundance of Cyanobacteria, from around 90% in the upper laminae to 20% in the lower laminae, over increasing depth within the cone was reported.

Comparing the pinnacle and conical mats, a distinct prokaryotic community structure was observed in pinnacle structure microbial mats. The increase in relative abundance of *Leptolyngbya* in the pinnacle structures is consistent with microscopy observations by Andersen et al. (2011). Vertical extensions into pinnacle structures are well documented in Antarctic environments and have been associated with the presence of *Leptolyngbya* (Sumner et al., 2016). Contrastingly, flat mats have been mostly linked with high abundances of *Phormidium* morphotypes, which are associated with a smoothing behavior that reduces small-scale topography and branching in mats (Mackey et al., 2015). Furthermore, Hawes et al. (2019) suggested the *Phormidium* morphotype filaments projecting from the top of the cones may represent a behavioral response to optimize carbon accrual at low DIC availability.

Microbial mat topography can often be linked to environmental conditions that influence biological processes related to metabolism, growth, motility and accumulation of biomass (Shepard and Sumner, 2010; Bosak et al., 2013). The occurrence of changing morphology of mat structures has been observed with changes along environmental gradients. For example, the differing morphology and subsequent community structure changes of Lake Fryxell corresponds with an oxygen gradient (Jungblut et al., 2016). Lake Untersee, however, provides an environment that is homogeneous along a depth gradient with respect to pH and oxygen saturation, and distinct structures clearly emerge within the same environment, at the same depth. PAR has often been suggested as an environmental factor that influences pinnacle formation through phototactic migration (Bosak et al., 2013). While there is some evidence of the influence of PAR on the structures of Lake Untersee (Andersen et al., 2011), with cones extending far beyond the depth of pinnacles that terminate at around 20 m, it is unlikely to be the sole determinator of stromatolite shape. The well-mixed water column of Lake Untersee is due to buoyancy-driven convection caused by the subsurface melting of the Anuchin glacier (Wand et al., 1997; Steel et al., 2015). Therefore, slight water movement occurs in the lake and may result in significant near-bed velocity gradients (Hawes et al., 2019). The impact of water movement on the structure morphology is currently unknown, it is possible that the presence of tall cones within moving water may increase nutrient supply to taller structures in the benthic communities over what would accrue on flats as they would be placed in higher velocity parts of the laminar flow gradient (Hawes et al., 2019).

The occurrence of distinct morphologies in sympatry therefore suggests that structure formation can be influenced by their associated microbial communities without external differences in the surrounding environment. The phylogenetic mapping of several cyanobacterial ASVs to sequences from the McMurdo Dry Valleys, particularly the pinnacles of Lake Vanda, suggests that the same morphotypes and potentially genotypes are involved in the formation of microbial structures in Antarctic lakes. The inherent behavior of cyanobacterial species has often been suggested to influence the formation of structures. It has been suggested that *Leptolyngbya spp.* is involved in vertical growth, and has been shown to form coniform structures in vitro (Reyes et al., 2013). Cell motility has been shown to be essential for the development of certain structures. Laboratory-based experiments have demonstrated that the unidirected gliding, colliding, and clumping of motile *Pseudanabaena* cells formed a biofilm with intersecting ridges, which developed into larger structures as cell numbers increased (Shepard and Sumner, 2010). While substrate and environmental conditions were seen to influence the ultimate structure, repeated structure formation was observed and associated with the self-organization of motile cells. A reduction in relative abundance of motile *Phormidium* may reduce the smoothing effect on the microbial mat structure and allow surface irregularities and pinnacle structures to form. Jones et al. (2002) showed that coniform stromatolites in hot springs only grew where the biota was dominated by *Phormidium*.

The mechanism underlying the morphogenesis of the large conical structures is currently unclear, as no distinct difference was seen in the prokaryotic community structure between flat and cone mat communities. Previous studies have suggested surface irregularities such as larger deposited grains or disrupted mat to initiate the emergence of structures (Andersen et al., 2011; Sumner et al., 2016) which is a plausible model for the morphogenesis of large cones. Hawes et al. (2019) also suggested that loose mesh of *Phormidium* morphotype trichomes on the top of the cones may increase trapping and enhance accumulation of sedimentation on the flattened apices of the cones, which might contribute to different levels of accumulation of mass on the top of the cones in comparison with the cone sides and the surrounding lake floor. These modern examples demonstrate the importance of microbial community diversity to the emergence of complex mat morphology. It is possible that ancient stromatolites were similarly shaped by diverse microbial communities and their behaviors and responses to their environment to produce structural differences on a spatial scale.

The contribution of non-cyanobacterial prokaryotic community members to structure morphogenesis is often discussed. For example, the metagenomes of microbialites of Cuatro Ciénegas, Mexico, were found to contain a large number of genes involved in sensory and regulatory systems as well as cell communication suggesting a coordination between cyanobacteria and heterotrophs (Breitbart et al., 2009). Additionally, evidence of viral-microbe interactions has been shown in microbial mat and stromatolite communities (Jarett et al., 2020; White et al., 2020). Viruses may contribute to structure morphogenesis through modulating microbial diversity and impacting microbial metabolism and ecosystem function through infection and the recycling of key nutrients.

The 18S rRNA gene data set did not show a clear difference in the microbial eukaryotic communities between cone, pinnacle, and flat mats. Whilst no differences were observed between different morphological structures, a vertical zonation in the mats was observed in the eukaryotic community structure with the loose mesh of filaments on the surface of the mats showing distinct clustering. A heterogenous distribution of eukaryotes has also been observed by Jackson et al. (personal communication), in cyanobacterial mat communities growing along the bottom of shallow meltwater ponds on the McMurdo Ice Shelf, Antarctica. Our data, therefore, do not provide evidence on how the eukaryotes may contribute to the formation of three-dimensional microbial structures and how individual taxa may influence appearance of specific mat morphologies in Lake Untersee. It is possible that the reduced presence of eukaryotes, particularly gliding diatoms, may allow the emergence of large conical structures that more closely resemble those of Early Earth. The impact of grazing pressure, particularly by larger rotifers and nematodes, is currently unknown, and may additionally influence the formation of large-scale structures.

## Conclusion

Lake Untersee is a unique environment that allows the formation of some of the best developed, microbially mediated, laminated sedimentary structures on modern Earth, and our study revealed that these macroscopic microbial mat communities harbor diverse cyanobacteria, bacteria and eukaryote communities. While the mechanism behind the morphogenesis of pinnacle and conical stromatolites is unclear, the results suggest that cyanobacterial species play a fundamental role. Our study of modern stromatolites has highlighted the importance of complex community structure to the formation of elaborate mat structures. Microbial metabolism, behaviors and interactions between bacterial and eukaryote members are likely to underpin structure morphogenesis, allowing distinct structures to form across a small spatial scale.

## Data Availability Statement

The high-throughput sequencing data and associated metadata from this study were submitted to the Sequence Read Archive (SRA) in GenBank under BioProject PRJNA638357.

## Author Contributions

DA and IH collected samples and collected environmental the data from Lake Untersee. IH and AJ designed the study. AB and CG processed the samples for sequencing. CG performed bioinformatics analysis and wrote the manuscript. MY and GB contributed to writing the manuscript. All authors contributed to and commented on the manuscript.

## Conflict of Interest

The authors declare that the research was conducted in the absence of any commercial or financial relationships that could be construed as a potential conflict of interest.

## Supplementary Material

Supplementary Table 1 Taxonomic assignments of prokaryotic ASVs.

Supplementary Table 2 Taxonomic assignments of eukaryotic ASVs.

Supplementary Table 3 BLAST assignment of cyanobacterial ASVs.
